# Assessment of Macular Parameter Changes in Patients with Keratoconus Using Optical Coherence Tomography

**DOI:** 10.1155/2015/245953

**Published:** 2015-05-12

**Authors:** Srujana Sahebjada, Fakir M. Amirul Islam, Sanj Wickremasinghe, Mark Daniell, Paul N. Baird

**Affiliations:** ^1^Centre for Eye Research Australia, East Melbourne, Vic 3002, Australia; ^2^The University of Melbourne, Parkville, Vic 3010, Australia; ^3^Department of Statistics, Data Science and Epidemiology, Swinburne University of Technology, Hawthorn, Vic 3122, Australia; ^4^Royal Victorian Eye and Ear Hospital, East Melbourne, Vic 3002, Australia

## Abstract

Keratoconus is typically diagnosed through changes at the anterior ocular surface. However, we wished to assess if macular parameter changes might also occur in these patients. We assessed posterior changes through the use of optical coherence tomography and compared to a nonkeratoconus patient group. All subjects underwent clinical examination including macular thickness measurements. The generalized estimation equation model was used to estimate the means and compare the differences in various measurements between keratoconus and nonkeratoconus patients. A total of 129 keratoconus eyes of 67 cases and 174 nonkeratoconus eyes of 87 controls were analysed. Keratoconus individuals presented with a significantly greater mean retinal thickness in the central fovea, inner, and outer macula compared to the nonkeratoconus group (*p* < 0.05). In addition, individuals presenting with the early signs of keratoconus had significantly greater inner and outer macular volume compared to the nonkeratoconus group (*p* < 0.05). This study indicates the retina appears to thicken at the fovea and macula and had increased macular volume in keratoconus individuals compared to nonkeratoconus individuals. Thus we posit that structural retinal changes exist in keratoconus eyes that are additional to those typically seen in the anterior segment.

## 1. Introduction

Keratoconus is a common corneal condition, typically having its onset in the teenage years and is characterised by a progressive corneal thinning that results in corneal protrusion, irregular astigmatism, and decreased vision [1]. The prevalence of keratoconus was estimated at 86 patients per 100,000 residents and the incidence at 1.3 per 100 000 per year [2]. In the early stages of keratoconus it is possible to correct a patient's vision with spectacles. With progression of keratoconus the patient's vision decreases and rigid gas permeable contact lenses are required to compensate for the optics of the irregular shaped anterior corneal surface. In a minority of patients, the central cornea becomes extremely thin and irregular and corneal transplantation surgery is required to restore vision [3]. Keratoconus is one of the most common indications for corneal transplantation, accounting for some 31% of corneal transplants in Australia [4] and 15.1% of corneal transplants performed in the United States [5].

Optical coherence tomography (OCT) is a powerful imaging technology allowing measurement of macular thickness changes. Typically such changes are used as an aid in disease diagnosis/progression, for diseases such as age-related macular degeneration (ARMD), diabetic retinopathy (DR), retinal vein occlusion, uveitis, and retinal dystrophies [6–13]. When this technique has been used for other ocular conditions such as myopia, studies have been inconsistent, with two Asian adult OCT studies in Japan and Singapore indicating that the average macular retinal thickness did not vary with refraction [14, 15]. However, in a separate study in Singaporean children, it was shown that the average macular volume and thickness were reduced with increasing myopia. In both the Singaporean adult and children's studies, the thickest point at the parafoveal region decreased with myopia, whereas foveal thickness increased [16]. There have been no reported studies showing whether macular thickness and volume changes also exist in other ocular diseases which affect the anterior portion of the eye such as in keratoconus.

In the current study, we wished to assess whether changes in posterior parameters, as measured by Stratus OCT (model 3000; software version 4.0, Carl Zeiss Meditec, International), also occurred in keratoconus subjects given that these individuals typically present with myopia due to corneal steepening.

## 2. Materials and Methods

All patients for this study were recruited from public clinics at the Royal Victorian Eye and Ear Hospital (RVEEH), private rooms (e.g., Eye Surgery Associates), optometry clinics (Lindsay Associates), or consenting general public with keratoconus. A patient information sheet, consent form, privacy statement, and patient rights were provided to all individuals participating in the study. Nonkeratoconus subjects were Europeans with mild refractive error obtained through the genes in myopia (GEM) study where a similar testing protocol was used [17].

### 2.1. Protocol

Keratoconus patients were required to complete a study questionnaire and undergo a complete clinical eye examination. The study protocol was approved by the Royal Victorian Eye and Ear Hospital Human Research and Ethics Committee (Project no. 10/954H). Written informed consent was obtained from each participant after explanation of the nature and possible consequences of the study. This protocol followed the tenets of the Declaration of Helsinki and all privacy requirements were met.

### 2.2. Inclusion and Exclusion Criteria

Individuals with keratoconus of European background, presenting to the clinics/private practices, were invited to participate in the study. Clinical keratoconus was diagnosed on the basis of the presence of one or more of the following [18–20]:

1. An irregular cornea, as determined by distortion of keratometric mires/and or computerized video-keratography.
2. Scissoring of the retinoscopic reflex.
3. Demonstrated at least one biomicroscopic sign, including Vogt's striae, Fleischer's ring, or corneal thinning and scarring typical of keratoconus.

And one or more of the following changes in topographic map

(a) focal steepening of areas greater than 47 diopters (D), located in the cone protrusion zone surrounded by concentric decreasing power zones,
(b) angling of the hemimeridians, exceeding 20 to 30 degrees, in the case of a bow tie pattern,
(c) inferior-superior asymmetry greater than 1.4 D within the mid peripheral cornea.

The results of flattest and steepest corneal curvature used in the study were from the four-map selectable display of Pentacam (Oculus, Wetzlar, Germany) results incorporating front and back elevation maps, along with front sagittal curve and pachymetry.

Potential subjects with nonkeratoconus ocular disease in both eyes such as keratectasia, corneal degenerations, macular disease, and optic nerve disease (e.g., optic neuritis, optic atrophy), and any other retinal changes were excluded from the study.

Pentacam and OCT images were obtained for the majority of the participants. Pentacam images could not be obtained from participants who came wearing contact lenses or from those who had undergone recent corneal surgery. Please refer to our recent publication, Sahebjada et al. (2014), for details of Pentacam measurements and results [21]. OCT could also not be performed on those with severe corneal scarring and recent corneal surgeries.

### 2.3. Eye Examination

The procedure involved collection of vision, objective and subjective refraction, slit lamp examination, and Pentacam examination which has been published elsewhere [21–24]. Axial length was recorded for each participant using a noncontact partial coherence interferometry with an intraocular lens (IOL) master (Carl Zeiss, Oberkochen, Germany).

### 2.4. OCT Measurement Procedures

An experienced operator performed the macular thickness scans through undilated pupils using Stratus OCT (model 3000; software version 4.0, Carl Zeiss Meditec, International). A fast macular thickness map scan protocol was followed to automatically obtain 3 consecutive macular scans, 6 mm in length, centred on the fovea, at equally spaced angular orientations. Scans were performed using the default axial length (24.46 mm) and refractive error (0.0 D) and manually adjusted to “*K*” readings of an individual (as per manufacturer's recommendations) for consistency with usual clinical practice. Although one can manually input the patient's axial length and refractive correction (Spherical Equivalent), these have no impact on magnification during scanning as the scan length in the fast/standard macular thickness scanning protocols cannot be adjusted in the Stratus OCT. Scans were accepted if free of artefacts (boundary errors and decentration), and complete cross-sectional images were seen for all individual line scans. After image acquisition, all macular images were manually checked to ensure that the foveal depression was evident in the centre of the scan. Up to 3 scans for each eye were obtained, and a single scan with the best quality per eye was selected for macular thickness analysis. Only scans with signal strengths of at least 5 out of 10 were used. In addition, a retinal specialist (ophthalmologist) carefully reviewed all scans for abnormalities, such as vitreoretinal traction, retinoschisis, and presence of lamellar macular hole.

Retinal thickness over the macula was automatically determined by the instrument software as the distance between the first signal from the vitreoretinal interface and the signal from the anterior boundary of the retinal pigment epithelium-choriocapillaris region. The retinal thickness/volume tabular protocol was selected as the analysis protocol, and the calculation of macular thickness was based on the 6 mm fast macular thickness map analysis printout. The map was composed of 9 sectors in 3 concentric circles, each with a diameter of 1 mm (central), 3 mm (inner), and 6 mm (outer circle), plus foveal minimum (the very central point of the fovea). The inner and outer regions were divided into 4 quadrants (temporal, superior, nasal, and inferior) and the average of the 4 quadrants was considered for analysis. The central circular region represented the foveal area. Foveal minimum thickness, means (standard deviations) of the thickness of central macula, and inner and outer regions by quadrants were presented. Subjects with severe corneal distortions wore their contact lens (except the ones that recently underwent corneal surgery) during the scans to avoid the astigmatic error (due to corneal asymmetry) that can affect the different line scans.

### 2.5. Statistical Analysis

Data from keratoconus and nonkeratoconus individuals were analyzed using SPSS software (version 21). The baseline characteristics of age were compared among the keratoconus and nonkeratoconus groups using One Way ANOVA and gender was compared using Chi square test. Spherical Equivalent (SE = sphere + half the cylinder) of refraction, corneal curvature in the flattest and steepest meridian (*K*1 and *K*2), axial length, and the OCT parameters were compared by Generalized Estimation Equation Model with post hoc *t*-test. Age, gender, SE, and corneal curvature were considered to be covariates.

## 3. Results

A total of 303 eyes from 154 individuals comprising 129 keratoconus eyes from 67 individuals [80 males (61.5%); mean age = 35 ± 13.5 years] and 174 nonkeratoconus eyes from 87 individuals [50 males (38.5%); mean age = 44 ± 14.9 years] were available for analysis. Compared to nonkeratoconus individuals, keratoconus subjects were significantly younger and more likely to be men (*p* < 0.001). The keratoconus eyes were significantly steeper and more myopic (*p* < 0.001) compared to the nonkeratoconus eyes. As such, these covariates were included in the analysis when comparing keratoconus and nonkeratoconus eyes for all OCT parameters, Table 1. There was no significant difference in the mean axial length (AL) between the 2 groups (24.1 mm versus 23.8 mm; *p* = 0.383).

Table 2 shows OCT characteristics of the keratoconus eyes compared to nonkeratoconus eyes after adjustment for the above covariates. The keratoconus group had significantly greater mean retinal thickness in the central fovea (CFT), inner (INT), and outer macula (OMT) compared to the nonkeratoconus group (*p* < 0.05). In addition the keratoconus group had significantly greater inner (IMV) and outer macular volume (OMV) when compared to the nonkeratoconus group (*p* < 0.005).

We also performed a subanalysis in only the early keratoconus eyes that had no evidence of corneal scarring/haze/opacities, average *K* (*K*1 + *K*2/2) ≤ 47.0 D, and did not wear contact lenses when the OCT was performed, Table 3. In this subanalysis, comparison of OCT parameters showed a significant difference only in the IMT and OMT between the early keratoconus and the nonkeratoconus eyes (*p* < 0.05).

## 4. Discussion

This study indicates that keratoconus patients have a thicker fovea and maculae and greater macular volume compared to nonkeratoconus individuals. This is the first study to investigate posterior segment changes in keratoconus subjects. Also our study suggests that inner and outer macular thickness are affected at the early stages of keratoconus subsequently followed by changes in the central fovea and macular volume as the keratoconus progresses towards more advanced forms of the disease. This may provide some indication of the natural aetiology of this disease in terms of effect at the retinal level. Also clinically, it may indicate that retinal examination of a patient offers a potential means of identifying individuals with keratoconus in conjunction with anterior changes assessment.

While it is unclear at present whether changes in macular parameters occur as a consequence of anterior changes, precede these changes, or are concomitant with anterior changes, we propose a couple of possible mechanisms. It is possible that our findings of increased foveal thickness potentially relate to the retinomotor movements of the photoreceptors, similar to those seen in form deprivation animal models, wherein it has been observed that the photoreceptor outer segments are elongated [25]. Perhaps in keratoconus eyes, the elongation of photoreceptors may be occurring as a compensatory change to the change in corneal curvature, although the exact mechanism is not clear. Further research needs to be conducted to prove this hypothesis. Also there may be retinal tissue growth happening in order to compensate for the thinning of the cornea and to prevent the general disorganization of the eye. Further studies of layer by layer analysis of retinal thickness with a spectral domain OCT may provide proof for this hypothesis.

While ideally corneal refractive surgery should not alter retinal structure, surprisingly Lei et al. reported an increase in total macular volume after Lasik [26]. Thus changes to the optical surfaces of the eye could be causing a systematic change in the apparent volume of retina scanned by OCT. This could be a compensatory mechanism within the eyes such that changes in one component have impact on another component.

Study strengths include the large sample size of keratoconus subjects. This is the first study to provide data of macular thickness and volume, using the Stratus OCT in a large population-based sample of Caucasian (European) keratoconus patients. Thus, these data provide a benchmark for clinicians to assess and compare normal and pathologic changes in the macula of keratoconus subjects. While the macular changes we identified in keratoconus patients have not been described before, they do suggest that other alterations are occurring in the eye aside from those in the anterior segment. While it is not yet clear if these changes precede or are concomitant with keratoconus, they may provide additional diagnostic modes of assessing early keratoconus in the clinic in other family members before other more obvious clinical signs occur. Firstly, OCT has become an important tool in the diagnosis and monitoring of various retinal disorders, such as ARMD, DR, retinal vascular disease, and glaucoma. The possibility of significant astigmatism, such as demonstrated in patients with keratoconus, may influence assessment of retinal thickness, a parameter used frequently in clinical trials, although commonly used OCT machines typically include a parameter setting for this. Secondly, the posterior segment changes may be related to the underlying disorganisation within the eye or may be a compensatory mechanism to minimise the effect of astigmatism. It would be interesting to see if there are changes in OCT parameters longitudinally to assess if there is progressive thickening or if treatment reverses these findings. A longitudinal study of keratoconus may help to answer this question and may help to define the optimal timing of treatment of keratoconus such as through cross-linking studies to prevent irreversible structural change. Finally, as there are significant changes in the inner and outer macular thickness in early keratoconus eyes using OCT, it would be interesting to see if these changes predispose to corneal curvature changes or vice versa.

There are a few limitations to the current study. The nonkeratoconus subjects that we used in the study had low myopia and defining what the “perfect control” is might have also included individuals classified with an emmetropic eye {an eye presenting with a refraction of between +0.5 D to −0.5 D}. However population studies indicate a skew from this position, either being slightly hyperopic or being present with substantial amounts of myopia [27]. Also, two Australian population-based studies have collectively estimated that approximately 20% of Australians aged between 40 and 60 years have myopia of equal to or worse than −0.50 D [28, 29]. Thus having nonkeratoconus subjects with low grades of myopia is representative of a substantial proportion of the general population in Australia. Additionally, none of the controls in the study had any corneal conditions and so can be considered to be corneal controls. Secondly, previous studies have documented that OCT measurements can be affected by segmentation error, which presents a challenge to automated algorithms [30]. Segmentation errors can be worse with weaker signals. However, we did not notice any consistent change in signal strength of OCT measurements among keratoconus and nonkeratoconus eyes. Also, while we attempted to minimise the potential impact of artefacts from sutured corneas in transplanted corneas, scarring or wearing contact lenses through assessment of only early keratoconus cases, assessment of individuals with corneal disease will always be difficult. Interestingly macular changes were noticed in individuals with early keratoconus who had not undergone any corneal surgery or had contact lenses on during OCT. Thus we do not believe that artefacts produced our results. However, the more pronounced macular changes noticed when the advanced cases were included may be because of the artefacts from the sutured corneas following graft or wearing contact lenses. While in retrospect these are potential weaknesses of the current study, they may be better controlled for through the use of spectral domain systems where there is more data to control placement of the volume estimate and better segmentation. Also we had a limited number of severe cases in our study. Having a larger number of eyes with advanced stages of keratoconus would have given a better picture of macular thickness and volume changes in this group which could then be compared to early keratoconus eyes. Further studies are therefore needed to confirm the current findings to establish whether macular parameter changes are always in this direction in keratoconus subjects. Another possibility may relate to default parameters used in OCT acquisition used by the Stratus instrument. A default axial length and refraction of 24.46 mm and 0.0 D are used in each OCT scan.

## 5. Conclusions

In summary, keratoconus patients appear to present with a thicker fovea and maculae and greater macular volume compared to nonkeratoconus individuals. This study suggests that important posterior segment differences exist in keratoconus eyes that are in addition to anterior changes. Such changes appear to occur in early disease and thus it would be of interest to assess members of keratoconus families who are currently undiagnosed with disease to assess whether these macular changes are evident. Used in conjunction with anterior surface changes, it may increase our prediagnostic ability in this group of individuals. Therefore macular changes may relate to a more generalised disorganisation of the eye in patients with keratoconus or a compensatory mechanism to optimise acuity in eyes with irregular corneas. However we do not know if these changes occur prior to or as a result of keratoconus. Further study is required to establish the clinical implications and reproduce our findings.

## Figures and Tables

**Table 1:** Baseline characteristics of study participants that were significantly different between the two groups (*p* < 0.001).

	Keratoconus eyes	Nonkeratoconus eyes
	(*N* = 129)	(*N* = 174)
	Mean ± SD	
Age (years)	35 ± 13.5	44.25 ± 15.0
Male %	62 (80)	38.5 (50)
SE (D)	−5.2 ± 0.45	−1.4 ± 0.36
*K*1 (D)	45.6 ± 0.45	43.4 ± 0.36
*K*2 (D)	51.3 ± 0.61	43.9 ± 0.49
AL (mm)	24.1 ± 0.24	23.8 ± 0.27

*N*: number of eyes.

SE: sphere + 0.5 Cylinder.

*K*1: flattest corneal radius.

*K*2: steepest corneal radius.

D: diopters.

AL: axial length.

mm: millimeters.

**Table 2:** OCT measures in keratoconus eyes compared to nonkeratoconus eyes.

Parameters	Group	Mean^∗^ ± SD	*p* ^∗∗^
CFT (*μ*m)	Keratoconus	220.7 ± 2.9	
	Nonkeratoconus	210.5 ± 2.3	***<0.05***

IMT (*μ*m)	Keratoconus	283.8 ± 1.9	
	Nonkeratoconus	273.8 ± 1.6	***<0.005***

OMT (*μ*m)	Keratoconus	246.6 ± 1.7	
	Nonkeratoconus	237.6 ± 1.4	***<0.005***

IMV (mm^3^)	Keratoconus	0.39 ± .003	
	Nonkeratoconus	0.38 ± .003	***<0.005***

OMV (mm^3^)	Keratoconus	1.35 ± 0.02	
	Nonkeratoconus	1.28 ± 0.01	***<0.005***

^∗^Mean adjusted for age, gender, SE, and corneal curvature.

^∗∗^ *p* values of keratoconus compared with nonkeratoconus.

CFT: central foveal thickness.

IMT: inner macular thickness.

OMT: outer macular thickness.

IMV: inner macular volume.

OMV: outer macular volume.

**Table 3:** Comparison of OCT measures between early keratoconus and nonkeratoconus eyes.

Parameters	Group	Mean^∗^ ± SD	*p* ^∗∗^
CFT (*µ*m)	Nonkeratoconus	210.5 ± 2.3	
	Early keratoconus (*N* = 85)	218.9 ± 4.3	0.2

IMT (*µ*m)	Nonkeratoconus	273.8 ± 1.6	
	Early keratoconus	281.5 ± 4.6	***<0.05***

OMT (*µ*m)	Nonkeratoconus	237.6 ± 1.4	
	Early keratoconus	244.6 ± 4.0	***<0.05***

IMV (mm^3^)	Nonkeratoconus	0.38 ± .003	
	Early keratoconus	0.37 ± 0.01	0.08

OMV (mm^3^)	Nonkeratoconus	1.28 ± 0.01	
	Early keratoconus	1.31 ± 0.04	0.21

^∗^Mean adjusted for age, gender, SE, and corneal curvature.

^∗∗^ *p* values of early keratoconus compared with nonkeratoconus.
